# Fine dissection of limber pine resistance to *Cronartium ribicola* using targeted sequencing of the NLR family

**DOI:** 10.1186/s12864-021-07885-8

**Published:** 2021-07-23

**Authors:** Jun-Jun Liu, Anna W. Schoettle, Richard A. Sniezko, Holly Williams, Arezoo Zamany, Benjamin Rancourt

**Affiliations:** 1 Canadian Forest Service, Natural Resources Canada, 506 West Burnside Road, Victoria, BC V8Z 1M5 Canada; 2 USDA Forest Service, Rocky Mountain Research Station, 240 West Prospect Road, Fort Collins, CO 80526 USA; 3 USDA Forest Service, Dorena Genetic Resource Center, 34963 Shoreview Road, Cottage Grove, Oregon, 97424 USA

**Keywords:** *Cronartium ribicola*, Limber pine (*Pinus flexilis*), NGS-based bulked segregation analysis (BSA), Resistance gene analog (RGA), Single nucleotide polymorphisms (SNPs), Targeted genomic sequencing (TS); white pine blister rust (WPBR)

## Abstract

**Background:**

Proteins with nucleotide binding site (NBS) and leucine-rich repeat (LRR) domains (NLR) make up one of most important resistance (R) families for plants to resist attacks from various pathogens and pests. The available transcriptomes of limber pine (*Pinus flexilis*) allow us to characterize NLR genes and related resistance gene analogs (RGAs) in host resistance against *Cronartium ribicola,* the causal fungal pathogen of white pine blister rust (WPBR) on five-needle pines throughout the world. We previously mapped a limber pine major gene locus (*Cr4*) that confers complete resistance to *C. ribicola* on the *Pinus* consensus linkage group 8 (LG-8). However, genetic distribution of NLR genes as well as their divergence between resistant and susceptible alleles are still unknown.

**Results:**

To identify NLR genes at the *Cr4* locus, the present study re-sequenced a total of 480 RGAs using targeted sequencing in a *Cr4*-segregated seed family. Following a call of single nucleotide polymorphisms (SNPs) and genetic mapping, a total of 541 SNPs from 155 genes were mapped across 12 LGs. Three putative NLR genes were newly mapped in the *Cr4* region, including one that co-segregated with *Cr4*. The tight linkage of NLRs with *Cr4*-controlled phenotypes was further confirmed by bulked segregation analysis (BSA) using extreme-phenotype genome-wide association study (XP-GWAS) for significance test. Local tandem duplication in the *Cr4* region was further supported by syntenic analysis using the sugar pine genome sequence. Significant gene divergences have been observed in the NLR family, revealing that diversifying selection pressures are relatively higher in local duplicated genes. Most genes showed similar expression patterns at low levels, but some were affected by genetic background related to disease resistance. Evidence from fine genetic dissection, evolutionary analysis, and expression profiling suggests that two NLR genes are the most promising candidates for *Cr4* against WPBR.

**Conclusion:**

This study provides fundamental insights into genetic architecture of the *Cr4* locus as well as a set of NLR variants for marker-assisted selection in limber pine breeding. Novel NLR genes were identified at the *Cr4* locus and the *Cr4* candidates will aid deployment of this R gene in combination with other major/minor genes in the limber pine breeding program.

**Supplementary Information:**

The online version contains supplementary material available at 10.1186/s12864-021-07885-8.

## Background

The development of genomic resources potentially offers new avenues for speeding the development of resistant populations for restoration of tree species affected by highly virulent pathogens. Several next generation sequencing (NGS) approaches have been developed and widely used for the identification of genomic regions of interest: including whole-genome sequencing (WGS), whole-exome sequencing (WES), and targeted genomic sequencing (TS) [1, 2]. Compared to WGS and WES, TS is a powerful approach that can fulfil the best balance between the accurate identification of targeted events with great sensitivity, and the overall cost and data burden for large-scale executions [3]. TS requires genomic DNA enrichment through either amplicon or capture-based hybridization. Because most plant disease resistance (R) genes encode proteins containing nucleotide-binding site (NBS) and leucine-rich repeat (LRR) domains (NLRs) or leucine-rich repeat receptor-like protein kinases (LRR-RLKs) [4], plant genomic regions encoding NLR proteins are attractive targets of TS. As one TS approach, resistance gene enrichment sequencing (RenSeq) has been used for improving genome annotations and genetic mapping of plant NLR genes [5, 6], the prioritization of novel NLR genes [7, 8], and identification of candidate R genes [9, 10].

Limber pine (*Pinus flexilis*) is a keystone species in ecosystems of high elevation in western North America. However, it is highly susceptible to infection by *Cronartium ribicola*, a non-native, invasive fungal pathogen that causes white pine blister rust (WPBR) on native five-needle pines in North America. WPBR is also a serious forest disease in Europe and Asia, but to lesser extent due to a much longer history of co-evolutionary arms races between the pathogen and its host trees. Since its arrival in western North America in the early 1900s, WPBR has led to severe economic losses of several five-needle pine species, including limber pine. In past decades, screening and breeding programs have identified both major gene resistance (MGR) and quantitative disease resistance (QDR) against WPBR. These resistance resources have been employed in plantations and restoration plantings for enhanced resistance in native five-needle pines in both the USA and Canada [11, 12]. So far, four loci have been identified for MGR against WPBR, including *Cr1* to *Cr4* in sugar pine (*P. lambertiana*), western white pine (*P. monticola*), southwestern white pine (*P. strobiformis*), and limber pine, respectively, in the USA [13–16]. *Cr4* has also been confirmed in seed families in Canada [17]. WPBR remains a devastating forest disease and continues to threaten successful restoration of limber pine and other five-needle pines in North America. Limber pine has been designated as an endangered species by the Government of Alberta and the Committee on the Status of Endangered Wildlife in Canada [18, 19].

Recent advances in NGS technologies and other related genomics approaches have been applied to understand the genetics of host resistance to *C. ribicola* for acceleration of the breeding cycle of five-needle pines. RNA-seq-based de novo transcriptome assembly and comparative profiling uncovered global gene expression and identified differentially expressed genes (DEGs) during white pine-blister rust (WP-BR) interactions, and annotation and interactions of these genes in various biological processes portraying the molecular mechanisms underlying tree defense responses and disease resistance of five-needle pines [20–23]. Whole genome sequencing of sugar pine (*P. lambertiana*) comprehensively revealed the organization and architecture of a very large conifer genome [24], providing an essential resource for the capture of genome-wide variations (such as single nucleotide polymorphisms-SNPs) for further genomic research and breeding programs [12, 25]. High-density genetic maps were developed for several species of five-needle pines, including sugar pine by SNP-genotyping arrays and WGS [12, 26], foxtail pine (*P. balfouriana*) by restriction site associated DNA sequencing (RADseq) [27], and limber pine by WES [28]. SNPs associated with QDR to *C. ribicola* in sugar pine were shown to be involved in wide biological functions, including disease resistance and morphological and developmental processes, by a combination of genome-wide association study (GWAS) and quantitative trait locus (QTL) analysis [12].

*Cr1*, *Cr2*, and *Cr4* were localized on the *Pinus* consensus LG-2, LG-1, and LG-8, respectively [21, 26, 29]. A combination of linkage mapping and association study validated *Cr4* or a locus very close to *Cr4* for limber pine MGR in seed families that originated in both USA and Canada [30]. These comparative studies of syntenic genomic regions of closely related species identified NLR genes as R candidates, which serve as good starting points for the positional cloning of five-needle pine R genes against *C. ribicola* [24, 31]. Although these R genes have been mapped, no R gene has been functionally characterized in five-needle pines. It is still unknown how each activates defense responses for resistance against *C. ribicola* in five needle pines.

Unlike *Cr1* and *Cr2* loci, few R gene analogs (RGA) of the NLR and RLK families were found to be clustered in the *Cr4* locus [28], hampering molecular study of disease resistance in this endangered conifer species. There have been few studies on the RGA families in conifers [32, 33]. Consequently, comprehensive analyses of the relationships between RGAs and host resistance to WPBR are indispensable. The present study used a Fluidigm amplicon-based TS approach to re-sequence resistance gene analogs (RGAs) to search for new candidate R genes for further investigation and deployment in limber pine breeding programs for the improvement of host resistance to *C. ribicola*.

## Results

### Targeted sequencing and SNP calling

Fluidigm custom access arrays were designed for 480 RGASs, which were selected from a limber pine transcriptome shotgun assembly (TAS accession no. GHWC00000000.2), for construction of MiSeq libraries using 96 genomic DNA samples (Table S1). Following adapter trimming and quality control, Illumina MiSeq generated a total 14.9 million 250-bp PE reads with high-quality, averaging 155 ± 22 thousand (K) reads per sample, with a range of 73 K ~ 206 K PE reads for individual samples (Table S2). Amplicon lengths of exonic sequences ranged from 250-bp to 350-bp, and amplicons in a total length of 161,333-bp were re-sequenced (Table S2). Mapping of the clean MiSeq PE reads to the reference gene sequences of the 480 RGAs showed 457 of them (95.2% of the total targets) were re-sequenced across the mapping population. A total of 2180 SNPs in 308 genes showed minor allele frequencies (MAF) > 5% across the mapping population. After filtering at MAF ≥ 0.3, 967 SNPs distributed in 277 genes were kept for further analyses (Fig. S1).

These polymorphic genes revealed SNP frequencies ranging from 2.8 SNPs to 52.5 SNPs per Kb (Fig. S2), indicating that a large part of the limber pine *R* gene families were highly polymorphic in the seed family LJ-112. The highest number of SNPs was found in the M428660 gene, and its available sequence encoded a toll/interleukin-1 receptor (TIR) domain. Eight others had high levels of polymorphisms > 40 SNPs/Kb. It would be interesting to know if high levels of genetic polymorphism of the limber pine NLR genes reflect their evolutionary adaptation to abiotic or other biotic factors than *C. ribicola*, since limber pine was not previously exposed to WPBR prior to the last century.

Plotting SNP depth against the total SNPs in individual samples showed that about 90% of SNPs had a minimum depth of 10 times in 91 samples (Fig. S3). The remaining four and one samples had about 70 and 15% of total SNPs with a minimum depth of 10 times, respectively (Fig. S3); these five samples were excluded in the 1st run for Lep-MAP 2, but added in the 2nd Lep-MAP 2 run for SNP mapping. Plotting missing data across the mapping population revealed that over 80% of total SNPs had missing data in less than 10% of total samples (Fig. S4). These results demonstrated that targeted re-sequencing by the Fluidigm custom access array-based MiSeq was effective for SNP discovery and detection in R gene families of conifer species such as limber pine.

### Genetic mapping of limber pine RGAs

**Fig. 1:**
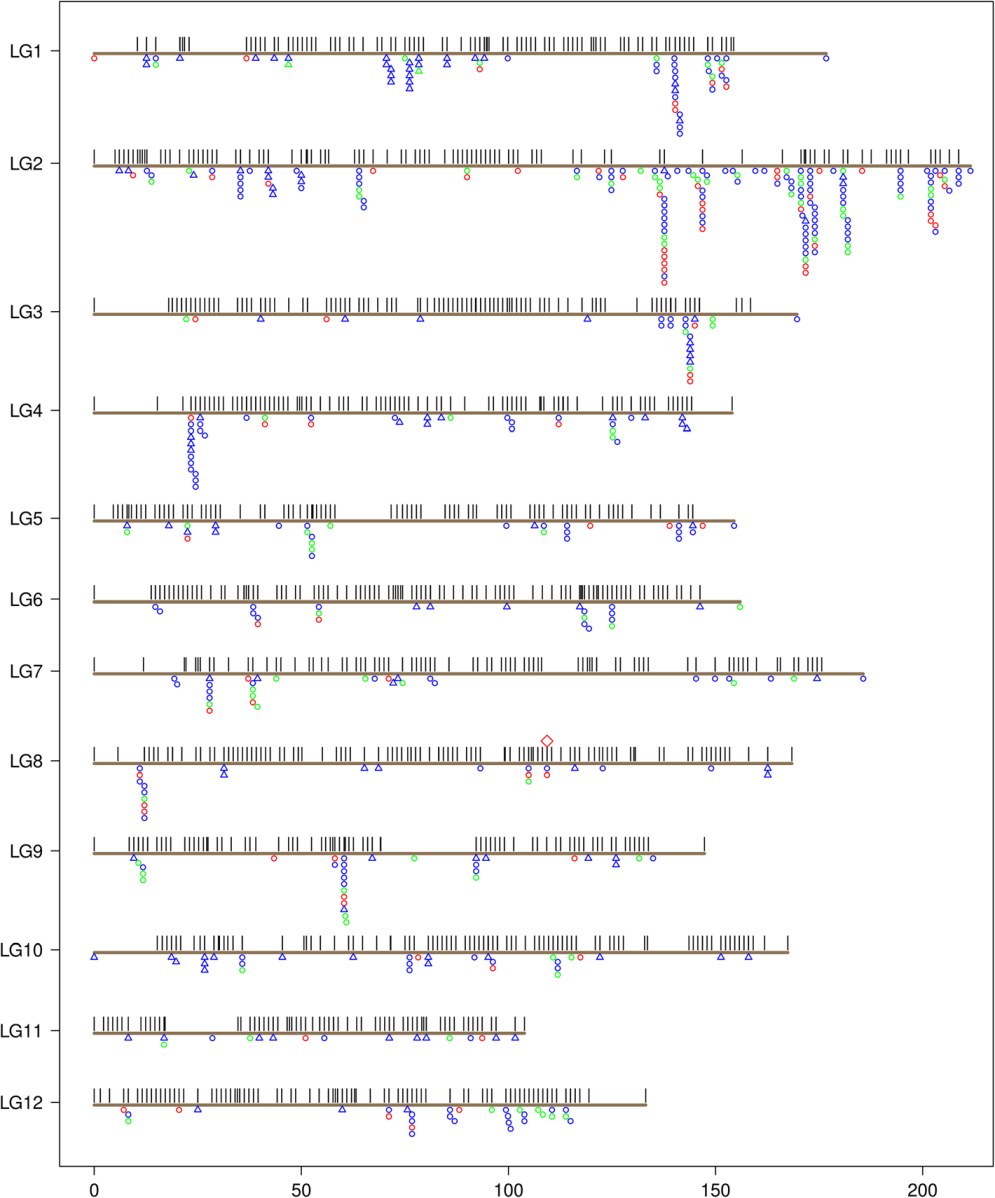
Genetic map of limber pine linkage groups (LGs) to show NBS-RR and RLK genes positioned in seed family LJ-112. Horizontal gray lines represent all 12 LGs. The x-axis represents LG length in centiMorgans (cM) and the y-axis indicates LG numbers. Black bars indicate the relative gene/marker positions, and circles and triangles below each LG indicate the positions of putative NLR and RLK genes, respectively. Genes mapped by either amplicon-based TS, WES, or both approaches are shown in colors of red, blue and green, respectively. The *Cr4* locus on LG-8 is represented by a diamond symbol

SNPs were filtered for missing data at 10% and high distortion from the expected Mendelian segregation ratio of 1:1 at α ≤ 0.01, generating 728 SNPs of 217 polymorphic genes for genetic mapping (Table S2). These SNPs were combined with other DNA markers from previous studies [21, 28] for Lep-MAP 2 runs. Among the 480 RGAs targeted by Fluidigm amplicons, a total of 541 SNP loci from 153 NLR and 2 LRR-RLK genes were mapped across 12 LGs (Table S3). With integration of previously mapped genes, genetic maps positioned a total of 5090 genes, including 387 putative NLR genes and 121 putative RLK genes in seed family LJ-112 (Fig. 1; Table S4).

**Fig. 2:**
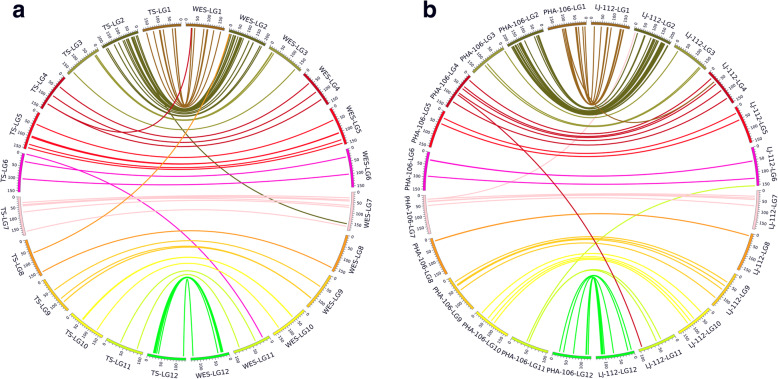
Comparison of genetic maps with NLR genes genotyped by different mapping approaches. Locations of bridging genes mapped by both TS and WES are shown by software Circles. The letters and numbers outside the circle represent linkage groups (LG), seed families, and mapping approaches, respectively. (a) Comparison of TS and WES in seed family LJ-112; (b) Comparison of TS and WES between seed family LJ-112 and PHA-106

Because the same reference transcriptome as described above was used in SNP calling, SNPs were directly compared for their types, nucleotide positions, and genetic mapping locations on the LGs between WES and Fluidigm amplicon-based TS. Compared to genes previously mapped by WES in the seed family LJ-112 [28], 79 additional genes were newly mapped in this study, and the remaining 76 genes were mapped by both WES and Fluidigm amplicon-based TS. Of the 76 genes mapped by both methods, SNPs of 72 genes (94.74% of total) were consistently mapped on the same LGs, at the same position or positions close to each other (Fig. 2a, Table S4). Of the other four genes (M581704, M598181, M604198, and M614586), SNPs aligned to the same gene were mapped on different LGs.

Genetic maps from two different seed families (LJ-112 and PHA-106) also showed similar consistency. Of 155 genes mapped here in family LJ-112, 82 genes were mapped previously by WES in family PHA-106 [28]. Paired SNPs of 78 genes (95.12% of the total) were mapped on the same LGs, while SNPs of four other genes (M332096, M507107, M604198, and M614454) were mapped on different LGs by the two mapping approaches (Fig. 2b). The SNPs of M604198 were mapped on different LGs using WES vs. Fluidigm approaches in LJ-112, as well as between LJ-112 and PHA-106. Thus a total of seven genes with paired SNPs were mapped on different LGs, compared to 148 mapped on the same LGs. These comparative maps demonstrated that both Fluidigm amplicon-based TS and WES are very effective for limber pine genetic mapping, with a high consistency of ~ 95% of total mapped genes between them (Fig. S5). For the seven genes mentioned above with paired SNPs on different LGs, the original physical distances between the paired SNPs were significantly longer than SNPs that mapped on the same LGs (928 ± 185-bp vs. 260 ± 37-bp in LJ-112; 1130 ± 167-bp vs. 311 ± 34-bp between LJ-112 and PHA-106, t-test *p* < 0.001) (Fig. S6). The physical distances of these misaligned SNP pairs were far outside the amplicon lengths as designed by Fluidigm-based PCR, suggesting that the SNP pairs of the same reference genes mapped on different LGs might have targeted paralogs with high nucleotide identities.

### Fine dissection of the *Cr4* locus and identification of *R*-candidates

**Fig. 3:**
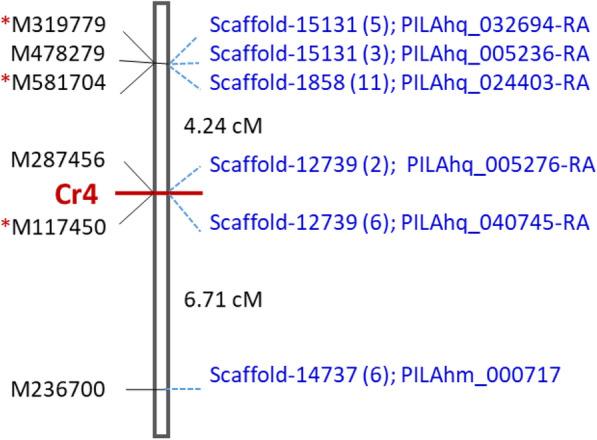
Fine genetic map of the limber pine *Cr4* locus on the *Pinus* consensus LG-8. Positions of six putative resistance gene analogs (RGAs) are shown, three NLR genes mapped by TS are labeled with red stars, and three others mapped previously by WES are included. The genetic distances between RGAs are represented by the scale in centiMorgan (cM) on the right. Sugar pine genome scaffolds and transcripts are shown on the right corresponding to orthologous genes of limber pine. Numbers of BLASTn-hit regions (including one orthologous region) inside the corresponding sugar pine scaffolds are indicated in parentheses

Of 155 RGAs newly mapped by TS in this study, three putative NLR genes (M117450, M319779, and M581704) were localized in the *Cr4* region on the *Pinus* consensus LG-8 with two SNPs of each gene. M117450 co-segregated with *Cr4* while M319779 and M581704 were localized within 4.45 cM of *Cr4* (Fig. 3). The tight linkage to *Cr4* was further confirmed by bulked segregation analysis (BSA) by comparing allele frequencies between bulked resistant and susceptible samples. Compared to genetic mapping, significance testing using an extreme-phenotype genome-wide association study (XP-GWAS) detected more genes and SNPs significantly associated with the resistance phenotype, with nine, five, and two SNPs in M117450 (2.24E-05 ≥ *p* ≥ 4.90E-15), M581704 (1.16E-06 ≥ *p* ≥ 8.04 E-07), and M319779 (6.49E-20 ≥ *p* ≥ 9.26E-20), respectively. Although NLRs M257518 and M350981 were not genetically mapped, their SNPs also showed significant association with *Cr4*-controlled phenotypes (1.16E-06 ≥ *p* ≥ 8.04E-07, 1.69E-04 ≥ *p* ≥ 8.75E-05; respectively), but significance levels were much lower compared to M117450 and M319779 (Fig. S7). In addition, two NLR genes (M287456 and M478279) and one RLK gene (M236700) were mapped on LG-8 by WES previously [28], with M287456 at 0.001 cM to C*r4*. Of six RGAs mapped in the *Cr4* region in seed family LJ-112 (Fig. 3), SNPs of M117450 and M287456 were further confirmed for their alleles in individual seedlings of families LJ-112 and four other MGR families using diploid needle samples by TaqMan arrays (Table S5).

### Fine genomic dissection of RGAs at the *Cr4* region

To evaluate the relationship of genetic and physical distances, as well as the complexity of RGA clusters in the *Cr4* region, all RGAs closely linked to *Cr4* were anchored to the sugar pine genome sequences (v1.5) by syntenic analysis using BLASTn. Of six RGAs in the *Cr4* region, one orthologous fragment was detected in the corresponding scaffolds of the sugar pine genome (Fig. 3). In addition, the same scaffolds were detected with paralogous fragments of multiple copies in a range from one (M287456 vs. scaffold_12739) to ten (M581704 vs. scaffold_1858) (Table S6). Most copies appeared to be pseudogenic gene segments.

M117450 and M287456 were mapped at almost the same position (0.001 cM genetic distance) independently by TS and WES approaches. Consistently, their corresponding orthologous regions were detected in the same scaffold (scaffold_12739) with 23.5 Kb physical distance as aligned to the sugar pine genome draft sequences (Fig. 3). This calculated as 23.5 Mb per cM in the *Cr4* region. BLAST search against sugar pine transcriptome showed that M117450 had the highest nucleotide identity of 93% to PILAhq_040745-RA, followed by 90% nucleotide identity to PILAhq_005276-RA, while M287456 had the highest nucleotide identity of 79% to PILAhq_005276-RA. Both sugar pine genes encode putative TNLs. The available sequence of M117450 covered both NBS and LRR domains, and had 88% amino acid identity to PILAhq_040745-RA. In contrast, the M287456 available sequence spanned a LRR domain region, and had 66% amino acid identity to PILAhq_005276-RA. Alignment of amino acid sequences revealed 30% identity between M117450 and M287456. These data indicated that M117450 and M287456 were different genes duplicated locally with high sequence similarity. In addition to orthologous regions, six other regions were detected as paralogs of M117450 and M287456 in sugar pine scaffold_12739, which spanned over 393-Kb. Similarly, M319779 and M478279 were mapped close to *Cr4* at the same position of LG-8 by WES and TS, respectively. Their orthologous sequences were only 1.5-Kb apart in sugar pine scaffold-15131.

Two SNPs of M581704 (890R and 1036S at nucleotide positions 890 and 1036, respectively) were mapped at the *Cr4* region of LG-8 by Fluidigm amplicon-based TS, but another SNP (120S at nucleotide position 120) of M581704 was previously mapped on LG-2 by WES (Fig. 2a; Table S4). This inconsistency was well explained by BLASTn analysis. The M581704 region positioned at 349 ~ 1134, (covering SNPs 890R and 1036S) had sugar pine scaffold_1858 as the top BLAST hit with 11 homologous regions in a range over 3 Mb, showing 94% nucleotide identity and 92% amino acid identity to the sugar pine transcript PILAhq_024403-RA. However, the M581704 region positioned at 1 ~ 379 (covering SNP 120S) had scaffold_6975 as the top BLAST hit with two homologous regions, showing 99% nucleotide identity and 98% amino acid identity to the sugar pine transcript PILAhq_010489-RA (Table S6). Putative proteins encoded by both PILAhq_024403-RA and PILAhq_010489-RA were annotated as NLRs based on BLASTp search against the NCBI-nr database. M581704 was a partial sequence encoding LRRs. High sequence identities of M581704 with both PILAhq_024403-RA and PILAhq_010489-RA across the highly variable LRR regions suggested that M581704 might be a fusion of two NLR paralogous genes that were erroneously jointed around the nucleotide positions 349 ~ 379. Genomic collinearity between limber pine and sugar pine genome assembly indicates limber pine NLRs were organized into clusters with multiple paralogs in the *Cr4* region. Moreover, each limber pine NLR was identified with multiple SNP loci from the fine genetic mapping, supporting their candidacy for *Cr4*.

### Phylogenetic and substitution analyses

**Fig. 4:**
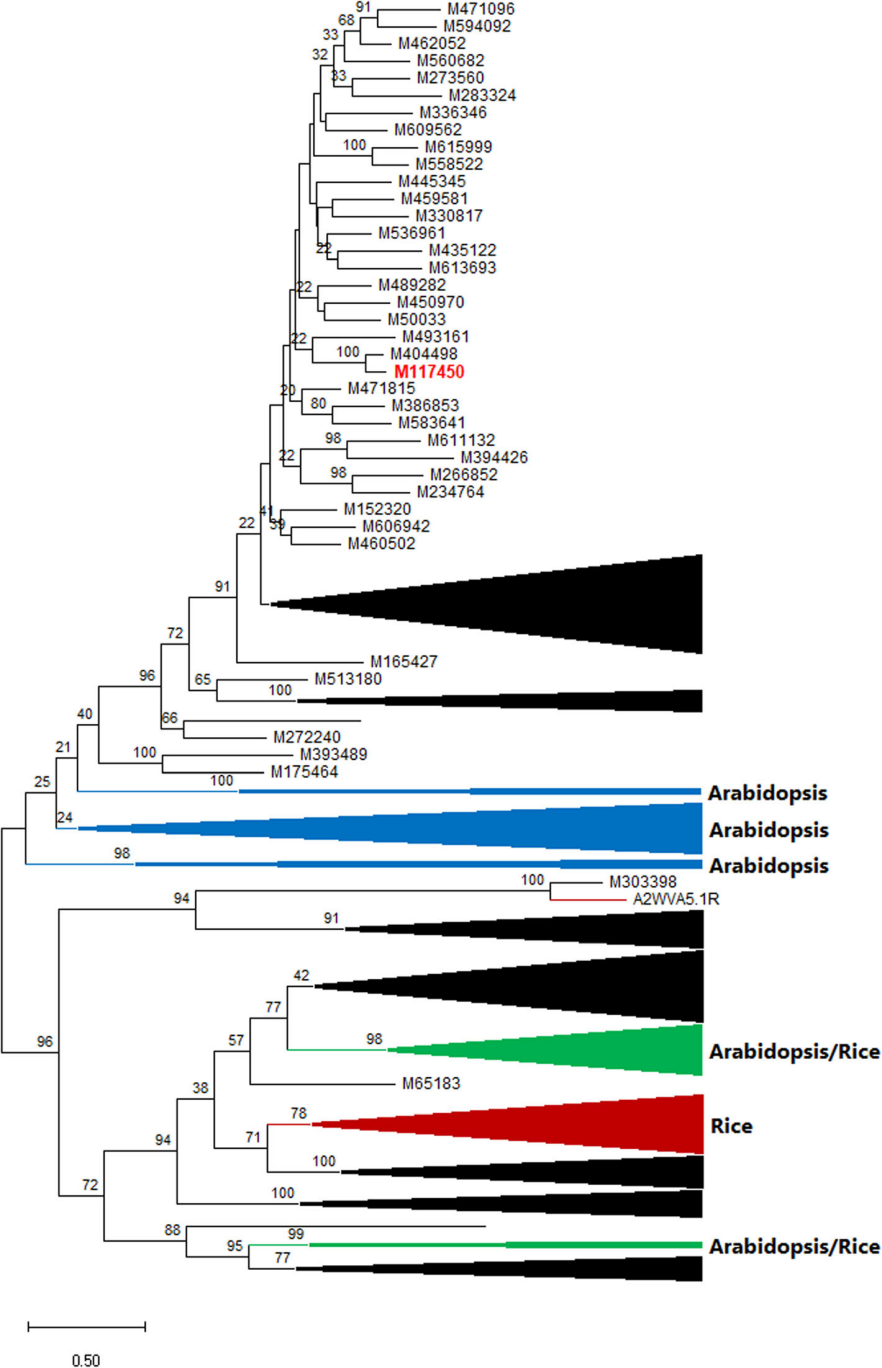
Phylogenetic tree of limber pine NLR family constructed using maximum likelihood (ML) method based on alignment of NB-ARC sequences. Arabidopsis and rice sequences that were shown as the top-hits in BLASTp as queried by limber pine sequences were included and labelled with UniProtKB accession numbers. A total of 158 limber pine NB-ARC sequences with a minimum length of 150 amino acids were clustered with 41 Arabidopsis and 27 rice NB-ARC sequences. The phylogenetic branches or clusters with sequences exclusively from limber pine, Arabidopsis, and rice are indicated in black, blue, and red, respectively. The phylogenetic clusters containing sequences from both Arabidopsis and rice are shown in green. Most cluster are collapsed while the cluster with M117450 (in red) as *Cr4* candidate is expended. Numbers near the nodes represent ML bootstrap values (> 20%)

DNA and putative protein sequences of all 9645 gene sequences so far genetically mapped in limber pine populations, including those mapped in this study, as well as those mapped previously by Sequenom- and WES-based SNP genotyping approaches [21, 28], are shown in Table S7. Of these sequences, 334 encode proteins with significant homologies (E-values < e-6) to available NB-ARC data sets by BLASTp analysis. Of these, 288 were further confirmed as having an NB-ARC domain (Pfam: PF00931) by HMM scan against the Pfam database, including 71 TS-mapped in this study and others retrieved from previous mapping studies. Putative NLRs without available sequence for NB-ARC confirmation, were annotated by presence of other NLR domains (such as TIR, Rx_N, RPW8, or LRR). Following removal of short sequences, 158 limber pine NB-ARC amino acid sequences were used for phylogenetic analysis to infer evolution of limber pine NLR family. The phylogenetic ML tree revealed that putative NLR proteins were divided into two main groups, corresponding to two NLR subfamilies that are well characterized based on their N-terminal features (Fig. 4). One group has an N-terminal domain potentially similar to the intracellular signaling domains of Drosophila Toll and the mammalian Interleukin-1 receptor (TIR), and are termed as TNL proteins. The other subfamily contains non-TNL members that commonly possess an N-terminal coil-coil (CC) domain, and is usually termed as CNL proteins. This branching pattern of the phylogenetic tree supports the hypothesis of ancient divergence of TNL and CNL subfamilies in plants. Limber pine TNL and CNL subfamilies were further divided into several clusters with deep divergence among them, indicating high evolutionary rates of NLR genes in this conifer species.

Five main clusters were observed in the CNL subfamily and strongly supported by the bootstrap test, four of which were embedded with at least one rice NB-ARC sequence, indicating their ancient origins before the separation of angiosperms and gymnosperms. In contrast, the limber pine TNL clusters were clearly separated from those of Arabidopsis proteins. No Arabidopsis NB-ARC sequences embedded in any cluster of the limber pine TNL subfamily, suggesting that limber pine TNLs expanded after angiosperms separated from gymnosperms. It is noteworthy that the TNL cluster harboring *Cr4*-co-segregated M117450 was the most complex with 32 NB-ARC sequences having long branches of divergence of up to 50% amino acid identity.

**Fig. 5:**
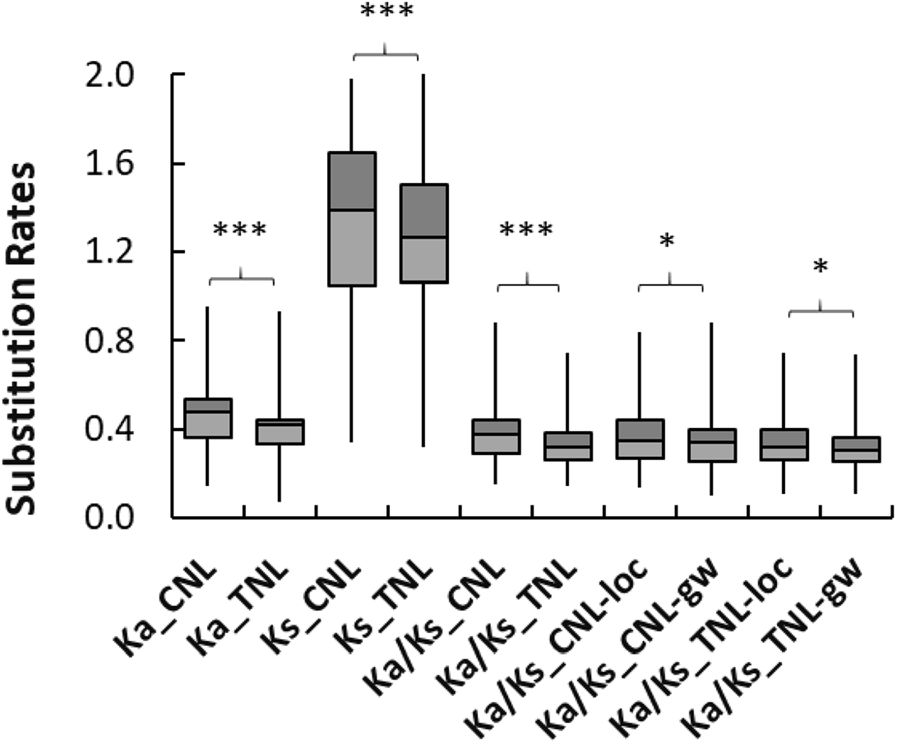
Box-plotting and comparison of substitution rates (Ka, Ks values and ratios of Ka/Ks) for limber pine NLR paralogous pairs determined by phylogenetic clustering

To detect the mode of selection, nucleotide substitution rates of nonsynonymous (Ka) and synonymous (Ks) sites and ratios of Ka/Ks were calculated for each paralogous pairs in the same clusters of the phylogenetic tree. Almost all paralogous pairs except two CNL pairs had Ka/Ks < 1 (Fisher test, *p* < 0.05), which indicated that most limber pine NLR genes (including M117450) were under purifying selection. Paralogous pairs of CNLs showed wider distributions of both the Ka/Ks ratios and values of either Ka or Ks compared to those of TNL paralogous pairs (Fig. 5). Mean values of Ka, Ks, and Ka/Ks ratios of CNLs were also higher than those of TNLs (Kolmogorov-Smirnov test, *P* < 0.001), suggesting that CNLs as a whole may have evolved earlier, but underwent relatively stronger diversifying selection and a faster evolutionary rate than TNLs in limber pine. Paralogous pairs of either TNLs or CNLs on the same LGs had Ka/Ks ratios significantly higher than pairs localized on different LGs (Kolmogorov-Smirnov test, *P* < 0.05) (Fig. 5).

### Expression of limber pine RGAs

**Fig. 6:**
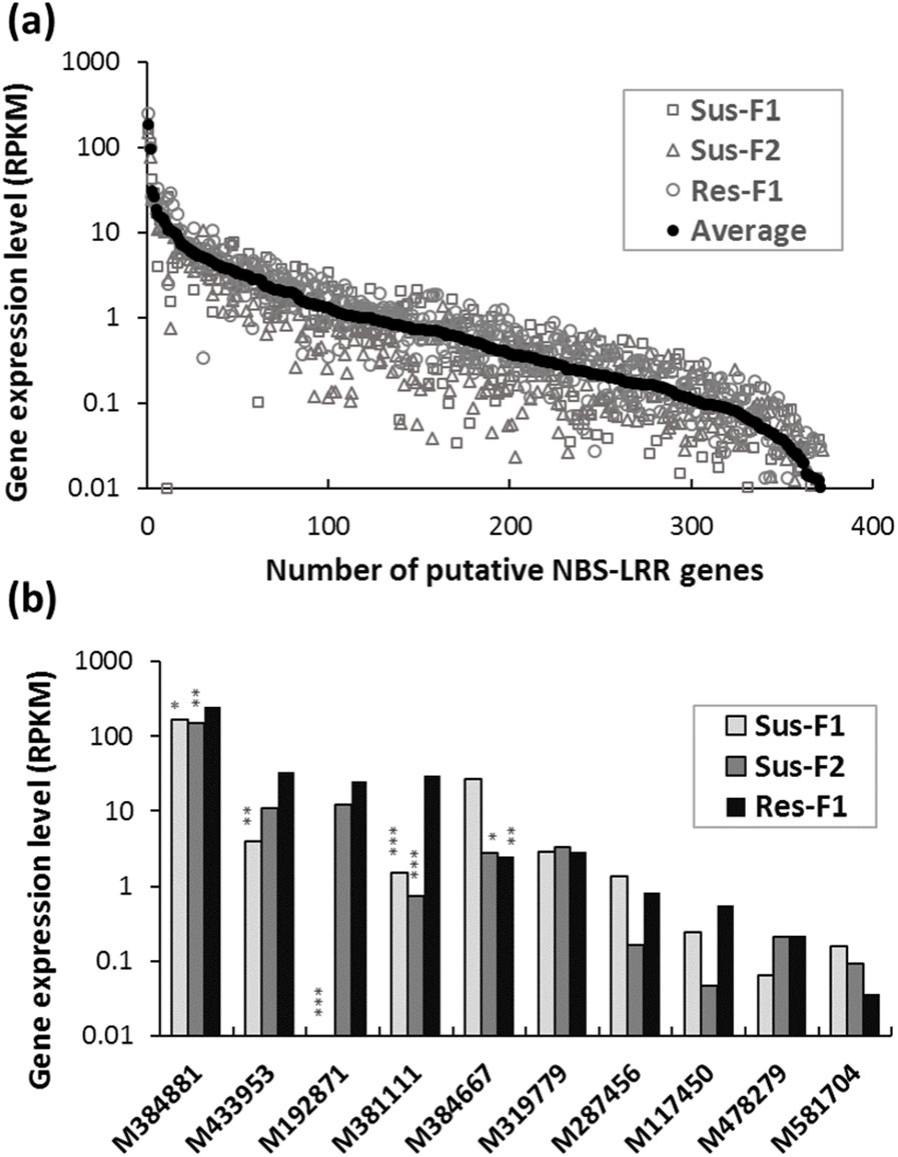
Expression level of limber pine NLR genes mapped in seed family LJ-112. Relative gene expression levels were calculated as Reads per Kilobase of transcript per Million RNA-seq reads (RPKM). (a) RPKM values (y-axis) are plotted across all 386 mapped genes (x-axis) with average value from the highest to the lowest. (b) Expression profiling of NLRs mapped at the *Cr4* region as well as genes with the differential expression across three seed families. Res-1: resistant seed family NR-3647; and Sus-1 and -2: susceptible seed families MRO-3501 and UT-3359A, respectively. Baggerley’s test was used to measure difference between seed families with FDR corrected *p*-values < 0.05 (*), < 0.01 (**), and < 0.001 (***)

To further evaluate the relationship between NLRs and disease resistance, expression patterns of limber pine NLRs were profiled using RNA-seq data, for three seed families with different genetic backgrounds: one resistant family (NR-3647) and two WPBR susceptible families (MRO-3501 and UT-3359A). Most NLR genes were expressed at low levels (RPKM < 5), including the five genes mapped in the *Cr4* region with RPKM in a range of 0.04 ~ 2.84 (Fig. 6a). Out of 386 putative NLR genes mapped in LJ-112, 45 genes were expressed at medium to high levels at RPKM ≥5 in at least one seed family while 14 showed no expression (RPKM = 0) in all three seed families with available RNA-seq data from needle samples. Most genes generally showed similar expression levels across the three seed families. Only five genes (M192871, M381111, M384667, M384881, and M433953) were detected with differential gene expression patterns across the three families, including four genes with higher expression in the resistant family than in at least one susceptible family (Fig. 6b). They were mapped on LG-4 (M192871 at 23.368 cM), LG-8 (M384667 at 11.013 cM, and M384881 at 12.126), LG-9 (M433953 at 60.328 cM), and LG-10 (M381111 at 117.374 cM), respectively (Table S5).

## Discussion

To search for candidate genes and new alleles for genetic resistance against *C. ribicola*, the present study applied an amplicon-based TS approach to re-sequence limber pine RGAs in a mapping seed family using the Fluidigm Access Array. Although several amplicon-based TS approaches are frequently used for SNP genotyping, few case studies have been reported that used Fluidigm Access Array in conifers [34]. The SNP calling results demonstrate that a large set of SNP data were rapidly detected using this amplicon-based TS approach, thus allowing us to identify novel alleles of the targeted genes in a conifer species with a huge, complex genome.

Compared with the WGS- or WES-based mapping approach, TS is one alternative approach to obtain genotypic data of specific genomic regions more accurately due to greater sequencing depth but with minimal cost [3]. TS thus has been used to sequence gene families and reduce genome complexities [35]. It has been applied to various research objectives, such as annotation of genomes, genetic mapping of new traits, and development of diagnostic tools to study the presence/absence of genes as well as sequence variations [5–8]. Despite exclusion of a proportion of SNP loci from genetic mapping due to their distortion from expected Mendelian segregation ratios, localization of SNPs still mapped one third of the targeted genes in a limber pine mapping population.

Of more than 300 R genes functionally well characterized in plants, the majority belong to the NLR family [4]. Therefore, in this study 474 NLRs and six RLKs were selected for re-sequencing, of which 155 were successfully mapped onto 12 LGs of seed family LJ-112, adding 69 new NLRs onto the genetic maps previously constructed. We revealed the high correlation and mapping accuracy by comparing gene positions on LGs between mapping approaches by amplicon-based TS and WES. The vast majority of SNPs, detected by TS and WES approaches independently in different mapping populations, showed high consistency of map positions for ~ 95% of the total mapped genes, indicating that most artifactual SNPs from alignment of paralogous sequences were excluded from genetic maps by multiple steps for SNP filtering as described in the methods. Furthermore, we used two different pipelines for qualitative and quantitative SNP calling, which identified the same set of TNL genes and alleles as *Cr4* candidates through both LG-based fine genetic mapping and NGS-based BSA with statistics of XP-GWAS. Non-consistent SNP pairs were observed in seven reference genes (~ 5% of total mapped genes) in comparative genetic mapping, which might be caused by gene fusion, or amplification of multiple paralogous sequences that all align to the same reference gene targeted by one pair of PCR primers [36]. The TS-based mapping accuracy and efficiency were similar to those of other NGS technologies (such as WGS, WES, and RADseq), or SNP array-based high throughput genotyping approaches in different conifer species [12, 26–28].

It is important to point out that the reference sequences we used for WES and Fluidigm amplicon-based TS were derived from transcriptome assembly of Illumina reads by the Trinity assembler, which is not an error-free process. Despite low levels of mis-assembly as compared to other short-read assemblers, mis-assembly rates of Trinity were 3.69% in Arabidopsis and 2.72% in human [37], but have not been reported in conifer species (including limber pine) yet. Multiple SNPs of the same reference genes consistently mapped by different NGS or other high-throughput approaches do not conclusively prove that their transcriptomic or genomic contigs are an accurate reflection of unique genes in the genome of an organism, especially for conifer species. De novo assemblies of conifer transcriptomes and genomes were more challenging than other plants due to large genomes with a high content of repeated sequences [23–25, 31, 33]. Once more accurate assemblers and SNP calling pipelines are developed, re-analyses of raw NGS data may provide more conclusive evidence to show the relationship between genetic and physical maps of a conifer genome. In a future study, cloning and Sanger sequencing of genomic DNA fragments would help clarification of potential mis-assembly of related RGA contigs, especially for M581704 in the *Cr4* region.

Host resistance to pests/pathogens is one of the most important focuses in plant breeding and conservation programs. Identification of functional candidates at R loci allows for comprehensive depiction of resistance mechanisms and evolutionary history, as well as development and application of DNA markers for marker-assisted or genomic selection. Characterization of the NLR family is a straight-forward avenue leading to genomic understanding of plant disease resistance in a species of economic or ecologic importance. Genome-wide NLR identification has in the past depended on gene predictions with available transcriptome and genome sequences. Previous studies mapped *Cr4* in a genomic region of LG-8 where only a short fragment of a putative NLR gene was mapped [28]. Most NLRs are present as gene clusters of different sizes, and only a small portion of family members stay alone as singletons in plant genomes [33, 38]. Despite the significant progress made so far on angiosperm NLRs, there are many unanswered questions related to evolution, regulation, and functional mechanisms of R genes [39, 40], and this type of knowledge is even more lacking in gymnosperms [32, 33]. This study mapped three more NLR genes in the *Cr4* region, with further verification by genotyping needle samples using TaqMan arrays and NGS-based BSA with significance test using XP-GWAS. These novel NLR genes and their variants added several *Cr4*-candidates to the *Cr4*-linked genes detected previously [21, 28]. Moreover, the putative NLR genes M117450 and M287456 each was identified with multiple SNP loci that co-segregated with *Cr4*-conferred resistance phenotypes with the fine genetic mapping.

Although most NLR family members were actively expressed at low levels, five genes were differentially expressed between resistant and susceptible seed families. Long noncoding RNAs (lncRNAs) affect a series of biological processes by targeting genes of various families with diverse functions in the transcriptional and posttranscriptional mechanisms of gene regulation [41, 42]. LncRNA-targeted genes included a large number of NLR genes [43, 44]. Regulation of NLR gene expression is rarely investigated in gymnosperms [32, 33], including five-needle pines [28, 44]. Genetic mapping excluded these five NLRs as *Cr4* candidates, but cannot exclude their potential roles in quantitative resistance against WPBR, or other pathogens as well as environmental stress [12, 28, 45, 46]. A recent RNA-Seq analysis indicated that the majority of differentially expressed NLRs are downregulated in response to drought stress [32], which is coincident with increased susceptibility to pathogens in stressed conifers [47, 48]. A future study on interactions of lncRNAs and their NLR targets in the limber pine transcriptome is required for better understanding of expression, function, and evolution of individual genes or alleles of this R gene family.

Of most limber pine NLR genes (including M117450 and M287456), constitutive expression at low levels avoids the fitness cost associated with disease resistance in the absence of pathogens [39]. Consistently, initial empirical results in limber pine suggests no constitutive cost of *Cr4* in the absence of WPBR [49]. *Cr4* appears to be in Hardy-Weinberg equilibrium in the absence of WPBR, suggesting that there is little to no current directional natural selective pressure on *Cr4* in these populations [16].

At present over 9600 functional genes have been genetically mapped in limber pine [21, 28]. Of these, 13% showed significant homologies (BLASTp E values < e-6) to Arabidopsis and rice NLR families and about 6% were further annotated as putative NLRs by BLAST2GO. Because of low expression levels, only partial sequences were available for a large part of NLR genes from limber pine transcriptomes. Of all mapped limber pine NLRs, 288 were confirmed by the presence of an NB-ARC domain detected using an HMM-scan against the Pfam database. Others had incomplete coding regions with only sequence features either upstream or downstream from the NB-ARC domain; thus some might represent discontinuous fragments of the same genes. Plant genomes usually encode a few hundred NLRs to defend against diverse and fast-evolving pathogens. The NLR family was identified with gene members numbering in a range of 338 to 725 in different conifer species [32, 33, 50]. The availability of complete genome sequence in the future will be needed to determine the size and genomic organization of the limber pine NLR family.

As expected, NB-ARC domain-based phylogenetic analysis divided limber pine NLRs into TNL and CNL subfamilies, with each further subdivided into several clusters. As one of the most promising *Cr4* candidates, M117450 resides in a TNL cluster with 32 paralogs. A comparison of Ka/Ks ratios suggests locally organized NLR paralogs might have stronger diversifying selection, with genes originating from tandem duplication. This hypothesis is further supported by a syntenic analysis between limber pine and sugar pine. Because the limber pine genome sequence is not available, we took advantage of the recent release of the sugar pine draft genome [24], and analyzed the position and similarity of the NLR genes in the *Cr4* region between these two closely related five-needle pine species, which are both highly susceptible to WPBR. At this point, five NLRs from the *Cr4* region had orthologs in three distinct sugar pine genomic scaffolds. Each orthologous pair showed the highest degree of similarity to the other, but low similarity to other gene family members, indicating their origin possibly prior to the divergence of the subgenus *Strobus*.

Genomic collinearity and the genetic architecture revealed by both fine mapping and syntenic analysis demonstrated that the *Cr4* locus contained an NLR gene cluster with high complexity. Strikingly, M117450 and M287456 are predicted to be very near to one another – their orthologs separated by an intergenic distance of only 23.5 Kb in the sugar pine genome. Meanwhile, the low level of sequence divergence with each other implied that they might have evolved by local gene duplication, not by genomic translocation of a different family member. This is not surprising because in a number of angiosperms recent expansion of the NLR family appears to have occurred mainly through tandem duplication rather than ectopic or segmental duplication [51, 52]. The duplication and diversification of NLRs in the *Cr4* region provide a potential for accumulation of mutations to create novel R genes or alleles, allowing limber pine to adapt its immune system against an ever-evolving rust pathotypes. During the dynamic “arms race” between plants and pathogens [53], expansion and rearrangement of gene members within genomic clusters is one of the main mechanisms for plants to adapt with new R genotypes [54, 55]. Although available evidence and evolutionary analysis support that M117450 and M287456 are the most promising R candidates, future functional investigation is needed to determine if one of them works alone or both interact with each other to act as *Cr4* for resistance against WPBR.

To restore WPBR-disturbed ecosystems at high elevations where limber pine is the keystone species, it is necessary to plant seedlings carrying a set of *R* genes or alleles against a spectrum of rust pathotypes. Undoubtedly, *Cr4* is a very valuable MGR locus that confers highly effective resistance to those tested pathotypes. However, breeding for resistance to WPBR is quite challenging in five-needle pines; MGR can be easily overcome by virulent isolates in field tests of western white pine and sugar pine [56, 57]. Although a virulent pathotype that defeats *Cr4* has not been discovered yet, extensive planting of limber pine using the *Cr4* genotypes over a long time period would accelerate the proliferation of virulent *C. ribicola* races should they evolve. Thus, there is an urgent need to identity novel R genes, especially those with broad-spectrum resistance, and QDR loci in the host trees as well as the pathotypes targeted by them, making it possible to breed five-needle pines carrying different R genes for sustainable WPBR management. Until these genetic resources are available, management strategies to minimize the proliferation of a virulent rust strain to *Cr4*, should recommend that limber pine planting stock include a mix of both *Cr4* and susceptible seedlings [58, 59].

The existence of *Cr4* in wide ranging geographical regions indicates its complicated genetic background [16, 17, 30], which limits *Cr4*-linked markers shared by different germplasms for development of molecular selection tools for wild stands. The present study detected sequence variations of *Cr4* candidate alleles, useful for tracing *Cr4* origin in limber pine populations. Genetic dissection of WPBR resistance would provide information and materials for a future study to develop tools and strategies for marker-assisted selection and genomic selection, facilitating the improvement of limber pine resistance to WPBR.

## Materials and methods

### Plant materials and phenotypic assessment

Seed trees of families LJ-112, GE213, CH125, CH130, and PS1383 were identified as limber pine by Dr. Anna W. Schoettle’s research team. Seeds were collected in 2003 and voucher seed samples are stored at USDA Forest Service, Rocky Mountain Research Station. The International Union for Conservation of Nature (IUCN) lists limber pine as ‘least concern’. USDA Forest Service researchers are allowed to collect limber pine seeds from non-listed species without a permit on federal lands. Experimental research and field studies on limber pine in this work, including seed collection, complied with relevant institutional, national, and international guidelines and legislation. Open-pollinated seed family LJ-112 was used for targeted amplicon sequencing of the limber pine R families encoding NLR proteins and RLKs. The seed tree of family LJ-112, from northern Colorado (40.79/− 106.49, elevation 2527 m a.s.l.), was previously identified with a heterozygous genotype (*Cr4/cr4*) for major gene (*Cr4*) resistance to *C. ribicola* by segregation analysis of the *Cr4*-controlled canker-free trait in its progeny populations [16, 21]. Megagametophyte tissues were collected individually from each seedling during seed germination in May 2014 at Dorena Genetic Resource Center (DGRC, Cottage Grove, Oregon). Seedlings were inoculated using *C. ribicola* basidiospores in September, 2014 at DGRC following a well-established protocol [16]. Following inoculation, WPBR disease symptoms were assessed for each seedling four times in January, February, April, and November, 2015. Phenotypes of 122 seedlings were determined for each seedling based on phenotypic segregation of stem-cankered and stem canker-free traits as described previously [16, 17], and 66% of them were resistant seedlings.

### Targeted amplicon sequencing using Fluidigm access array system

Haploid megagametophyte samples from 91 seedlings of seed family LJ-112 were used for targeted amplicon sequencing. Needle tissues of five *Cr4-*resistant seedlings, each from one MGR seed family (LJ-112, GE213, CH125, CH130, and PS1383), were included as diploid controls. Genomic DNA was extracted from megagametophyte and needle tissues using a DNeasy Plant Mini kit (QIAGEN). A set of 480 limber pine RGAs, including 474 NLR-encoding genes and six LRR-RLK-encoding genes (Table S1), were selected from a limber pine transcriptome shotgun assembly (TAS accession no. GHWC00000000.2) as re-sequencing targets. If there were multiple transcripts for a unigene in the shotgun assembly, the longest transcript was selected as the representative sequence, with reference to other limber pine TAS assemblies available from the GenBank in cases where longer open read frame (ORF) sequences were available. RGAs were annotated based on their homologies to NLR and RLK proteins in the available databases (NCBI-nr, PIR, KEGG, and GO) as revealed by using BLAST2GO [60]. BLAST analyses were used to explore homologies of limber pine genes to other conifers by searching against the genome sequences and the putative proteome of loblolly pine (*P. taeda*, 84,522 proteins) and of sugar pine (85,053 proteins) [24, 61]. Sugar pine genome sequences (v1.5) were used as references for syntenic analysis by BLASTn searches to obtain genomic information for RGAs selected from limber pine, including prediction of exonic regions of each limber pine gene. One exon per gene was used for the design of Fluidigm PCR primers (Table S2). The amplicons were designed based on the ORFs of the targeted genes. Amplicon-based libraries were prepared using the Fluidigm Access Array system by parallel amplification of 48 unique samples with the primers pooled at 10 pairs per well [62]. PCR was performed with 50 ng genomic DNA. Each sample was indexed for combination of amplicons to generate multiplexed libraries. Following purification, amplicon libraries were sequenced for 250-bp paired-ends (PE) using an Illumina MiSeq sequencer.

### MiSeq read mapping and SNP analysis

Illumina MiSeq reads were demultiplexed using the sample-specific barcodes and trimmed for removal of the Fluidigm Access Array barcodes with a quality score of 0.05. A SNP calling pipeline as described previously [28] was used to detect DNA variants. In brief, the reference files of targeted exon sequences was formatted using PICARD-TOOLS 2.3.0, rebuilt using BOWTIE2 2.2.9 [63]. MiSeq clean reads of each sample were aligned with the generated reference using BOWTIE2 2.2.9 with the arguments ‘local’ and ‘verysensitive-local’ [63]. The SAM files generated from read-mapping were converted to BAM files, and they were sorted and indexed using SAMTOOLS 1.3.1 [64]. Sequence variant detection and genotype calling were performed with the BAM files as input using FREEBAYES 1.0.2–16-gd466dde for haploid mode (ploidy = 1) run with default parameters, outputting VCF files [65]. Finally, VCFTOOLS 0.1.12b [66] was used to process DNA variant data in the VCF files. Only SNPs were analyzed and other variants (short indel, MNV, and presence/absence variants) were excluded in this study. SNP data from individual samples were merged and analyzed in tab-format files using in-house R scripts. Statistics for read mapping to reference sequences were checked for distributions of SNP depth, missing data, and MAFs for evaluation of potential errors from Fluidigm-based PCR, MiSeq, and read mapping before further genetic map construction.

### Genetic map construction

Based on SNP genotypes from the megagametophye population, haploid segregation analysis was used to map NLR genes. Mapping expressed genes in conifers is difficult due to the presence of paralogs and pseudogenes. To avoid SNPs called from paralogous sequences, SNP data were initially filtered by X^2^ test and a check for missing data prior to mapping analysis. Genotypic segregation of SNP loci was tested for Mendelian ratio of 1:1 by X ^2^ (α = 0.05). SNP loci were filtered by significant segregation distortion (*P* < 0.01) and missing data at 10% for initial genetic mapping analysis. Because over 80% of all the SNPs had missing data levels of less than 10%, SNPs with > 10% of missing data were added later in the mapping analysis. In addition to the SNPs of the NLR family detected in the present study, other DNA markers available from previous studies (Table S4 and S7), including markers and genes mapped by Sequenom’s MassARRAY genotyping [21] and exome-seq [28], were included in the mapping analysis for this seed family.

Lep-MAP 2 was used for genetic map construction as described previously [67]. In brief, DNA markers were assigned into LGs using the separate chromosomes module at lodLimit = 10, and other remaining SNP markers were added to existing LGs by the joinsingles module at lodLimit = 6. SNP loci were positioned within each LG using the ordermarkers module by maximizing the likelihood of the data given the order using input parameters alpha = 0.1, polishWindow = 100, filterWindow = 10, sexAveraged = 1. Most SNPs of the same genes were positioned at the same site of the LG in the first run of Lep-MAP 2. SNPs potentially called from alignment of paralogous sequences were further filtered during the mapping process; genes were removed from the final map construction if the first run of Lep-MAP 2 assigned multiple SNPs of the same reference sequences to different LGs. For the genes with multiple SNPs mapped in the 1st run of Lep-MAP 2, the SNP with the lowest missing data, the lowest error estimate and the closest position to the median position was chosen as the representative SNP and mapped in the 2nd run of Lep-MAP 2. Two separate linkage maps were initially constructed for the *Pinus* consensus LG-9 and they were assembled into one LG based on bridging genes mapped by WES previously in seed families LJ-112 and PHA-106 [26].

Because the same reference transcriptome was used, SNPs of the bridging genes mapped by both amplicon-based TS and WES were directly compared for LG localizations in the same (LJ-112) or different seed families (LJ-112 and PHA-106) and visualized using CIRCOS [68]. RGAs mapped on the same LGs by both TS and WES were further subjected to Pearson correlation analysis to check mapping consistency between TS and WES approaches.

### Verification of *Cr4*-linked SNP markers by bulked segregation analysis (BSA) and TaqMan assays

NGS-based bulked segregation analysis (BSA) [69] was used to verify *Cr4* candidates. The Res-pool has MiSeq reads from 52 resistant seedlings, and the Sus-pool had MiSeq reads from 39 susceptible seedlings. Clean reads were mapped to the reference of RGA sequences using CLC genomics workbench (v5.5) with setting at mismatch cost = 2, insertion cost = 3, deletion cost = 3, length fraction = 0.9, similarity fraction = 0.95, auto-detect paired distances = yes, global alignment = yes, and non-specific match handling = ignore. SNPs were called by quality-based variant detection with settings at neighborhood radius = 5, maximum gap and mismatch count = 2, minimum neighborhood quality = 15, minimum central quality = 20, ignore non-specific matches = yes, ignore broken pairs = no, minimum coverage = 4, minimum variant frequency (%) = 1.0, and maximum expected alleles = 2. A total of 5608 SNPs of 354 RGAs were detected in both Res-pool and Sus-pool with coverage > 50 in each pool, and used for the significance test using extreme-phenotype genome-wide association study (XP-GWAS) [70, 71]. The best *Cr4*-candidates were predicted with difference of allele frequencies close to 0.5.

*Cr4* candidates were selected for verification of their SNPs by TaqMan assays (Table S5). TaqMan assays were first verified using genomic DNA of megagametophyte tissues from LJ-112 and then tested using genomic DNA of needles. TaqMan-based SNP genotyping was carried out using a 7500 Fast Real-Time PCR system (Applied Biosystems) and following the procedure as instructed by the manufacturers.

### Gene expression analysis by RNA-seq

To analyze the expression of limber pine RGAs, the RNA-seq data of one resistant (NR-3647) and two susceptible seed families (MRO-3501 and UT-3359A) were downloaded from GenBank (SRA accession numbers SRR3273741-SRR3273743) [21]. Clean reads were mapped to the limber pine reference transcriptome with a minimum length fraction of 0.9 and a minimum similarity fraction of 0.9. Reads per Kilobase of exon per Million fragments (RPKM) were calculated as relative gene expression values using CLC Genomics Workbench 5.5 (CLC bio, QIAgen, Aarhus, Denmark). Baggerley’s test was used to measure difference of gene expression levels between seed families. False discovery rate (FDR < 0.05) was used to adjust for multiple testing.

### Phylogenetic and nucleotide substitution analyses of limber pine NLR genes

BLASTp analysis was used to determine domain presence of the mapped limber pine RGAs by searching against the NB-ARC domain dataset (Pfam: PF00931) downloaded from the UniProtKB sequence database [72] and further confirmed by HMMSCAN against the HMM database using an on-line server at the European Bioinformatics Institute (EMBL-EBI; https://www.ebi.ac.uk/Tools/hmmer/search/hmmscan). Predicted amino acid sequences of the conserved NBS domain (Pfam 00931) with a minimum length of 150-amino acids were aligned using Clustal Omega [73]. Based on sequence alignment, phylogenetic analysis was performed using Mega-X with the maximum likelihood method [74, 75]. In addition, *Arabidopsis thaliana* and *Oryza sativa* NB-ARC (PF00931) sequences were retrieved from the Pfam database at EMBL-EBI; and those sequences shown as top-hits when queried by limber pine NB-ARC sequences in BLASTp analysis were included in the phylogenetic analysis. Reliability of the interior nodes of the phylogenetic tree was evaluated by bootstrap analysis with 100 replicates. NLR clusters comprising multiple paralogous sequences were used to determine the synonymous (Ks) and non-synonymous (Ka) substitution rates for each paralog pair.

ParaAT (v 2.0) was used to align nucleotide and protein sequences for each pair of paralogs [76]. The aligned sequences were used to estimate Ks and Ka values, and Ka/Ks ratios using the KaKs Calculator software (v 2.0) with model averaging method [77]. The Fisher test was used to determine whether ratios of Ka/Ks are significantly different 1. Ka/Ks = 1, Ka/Ks > 1, and Ka/Ks < 1 indicated neutral, positive, and purifying selection, respectively**.**

## Supplementary Information

**Additional file 1: Fig. S1.** Distribution of minor allele frequency (MAF) of SNP loci detected in limber pine resistant gene analogs (RGAs). **Fig. S2.** SNP frequencies of limber pine resistance gene analogs (RGAs). **Fig. S3**. Sequencing depth of SNP loci in individual samples. The percentages of total SNPs (x-axis) have a coverage of a certain depth (y-axis). SNP depth was assessed in each individual of seed family LJ-112. The plot displays data for SNPs (967) with MAF ≥ 0.3 and about 70% of them have a minimum depth at 10 x in all samples except one. **Fig. S4.** The extent of missing data for 967 SNP loci in 96 individual samples of seed family LJ-112. Individual samples with missing data were calculated as a percentage of the total (y-axis) and plotted across the cumulative total SNPs (x-axis). Over 80% of total SNPs showed missing data in less than 10% of total individual samples. **Fig. S5.** Correlation of SNP positions of the same NLR gene mapped on the same linkage groups (LGs) by both Fluidigm amplicon-bases targeted-seq (TS) and whole exome-seq (WES). (a) Comparison of TS and WES in seed family LJ-112; (b) comparison of TS and WES between seed families LJ-112 and PHA-106. **Fig. S6.** Physical distances (bp) of paired SNPs mapped by Fluidigm amplicon-bases targeted-seq (TS) and whole exome-seq (WES). **Fig. S7.** Identification of NLR alleles significantly associated with MGR-conferred phenotypes using extreme-phenotype genome-wide association study (XP-GWAS). (a) Quantile–quantile plot of the test statistic: 5608 SNPs detected in 354 RGAs with coverage > 50 were subjected to association analysis. (b) Manhattan plot: top SNPs were selected for each genes and plotted against genetic maps of 12 linkage groups, and those genes not mapped so far were included as a separate group. Significant threshold value (*p* = 1.69 × 10^− 4^) is presented by a horizontal dash line.

**Additional file 2: Table S1**. PCR primers and gene sequences for amplicon-based targeted sequencing of limber pine resistance gene analogs (RGAs). **Table S2.** Summary of targeted-seq of limber pine RGAs and their SNPs detected for genetic map construction in the seed family LJ-112. **Table S3.** RGAs and their SNPs mapped by amplicon-based targeted genomic sequencing in seed family LJ-112. **Table S4.** Summary of functional genes and their representative SNPs genetically mapped in seed family LJ-112 in the present and previous studies. **Table S5.** Oligonucleotides designed for TaqMan-based SNP genotyping. **Table S6.** Syntenic analysis of *Cr4*-linked NLRs with sugar pine genome sequences. **Table S7.** DNA and protein sequences of functional genes so far genetically mapped in limber pine seed families.

## Data Availability

The datasets generated and/or analyzed during the current study, including sequences of DNA and proteins, genetic polymorphisms, and linked genotype and phenotype data, are available in GenBank under Bioproject accession no. PRJNA315892 with TAS accession no. GHWC00000000.2, as well as in the Supplementary files.

## Abbreviations

BSA: Bulked segregation analysis
CNL: Coiled-coil NBS-LRR protein
Ka/Ks: The ratio of the number of nonsynonymous substitutions per nonsynonymous site (Ka) to the number of synonymous substitutions per synonymous site (Ks)
LG: Linkage group
WES: Whole exome sequencing
MGR: Major gene resistance
NB-ARC: Nucleotide-binding adaptor shared by APAF-1, R proteins, and CED-4 domain
NGS: Next generation sequencing
NLR: Protein with domains of nucleotide binding site (NBS) and leucine-rich repeats (LRR)
QDR: Quantitative disease resistance
SNP: Single nucleotide polymorphism
RGA: Resistance gene analog
RLK: Receptor-like kinase
WPBR: White pine blister rust
TIR: Domains of drosophila toll and the mammalian interleukin-1 receptor (TIR)
TNL: TIR-NBS-LRR protein
TS: Targeted genomic sequencing
XP-GWAS: Extreme-phenotype genome-wide association study
