# Supplementary material for: Fine dissection of limber pine resistance to Cronartium ribicola using targeted sequencing of the NLR family
Source: BMC Genomics. 2021 Jul 23;22:567. doi: 10.1186/s12864-021-07885-8 (PMC8299668; doi:10.1186/s12864-021-07885-8)
Supplement: Supplementary file 1 — Additional file 1: Fig. S1. Distribution of minor allele frequency (MAF) of SNP loci detected in limber pine resistant gene analogs (RGAs). Fig. S2. SNP frequencies of limber pine resistance gene analogs (RGAs). Fig. S3. Sequencing depth of SNP loci in individual samples. The percentages of total SNPs (x-axis) have a coverage of a certain depth (y-axis). SNP depth was assessed in each individual of seed family LJ-112. The plot displays data for SNPs (967) with MAF ≥ 0.3 and about 70% of them have a minimum depth at 10 x in all samples except one. Fig. S4. The extent of missing data for 967 SNP loci in 96 individual samples of seed family LJ-112. Individual samples with missing data were calculated as a percentage of the total (y-axis) and plotted across the cumulative total SNPs (x-axis). Over 80% of total SNPs showed missing data in less than 10% of total individual samples. Fig. S5. Correlation of SNP positions of the same NLR gene mapped on the same linkage groups (LGs) by both Fluidigm amplicon-bases targeted-seq (TS) and whole exome-seq (WES). (a) Comparison of TS and WES in seed family LJ-112; (b) comparison of TS and WES between seed families LJ-112 and PHA-106. Fig. S6. Physical distances (bp) of paired SNPs mapped by Fluidigm amplicon-bases targeted-seq (TS) and whole exome-seq (WES). Fig. S7. Identification of NLR alleles significantly associated with MGR-conferred phenotypes using extreme-phenotype genome-wide association study (XP-GWAS). (a) Quantile–quantile plot of the test statistic: 5608 SNPs detected in 354 RGAs with coverage > 50 were subjected to association analysis. (b) Manhattan plot: top SNPs were selected for each genes and plotted against genetic maps of 12 linkage groups, and those genes not mapped so far were included as a separate group. Significant threshold value (p = 1.69 × 10− 4) is presented by a horizontal dash line. [file 12864_2021_7885_MOESM1_ESM.pdf]

The title of the manuscript: Fine dissection of limber pine resistance to *Cronartium ribicola* using targeted sequencing of the NLR family

The authors: Jun-Jun Liu, Anna W. Schoettle, Richard A. Sniezko, Holly Williams, Arezoo Zamany, Benjamin Rancourt

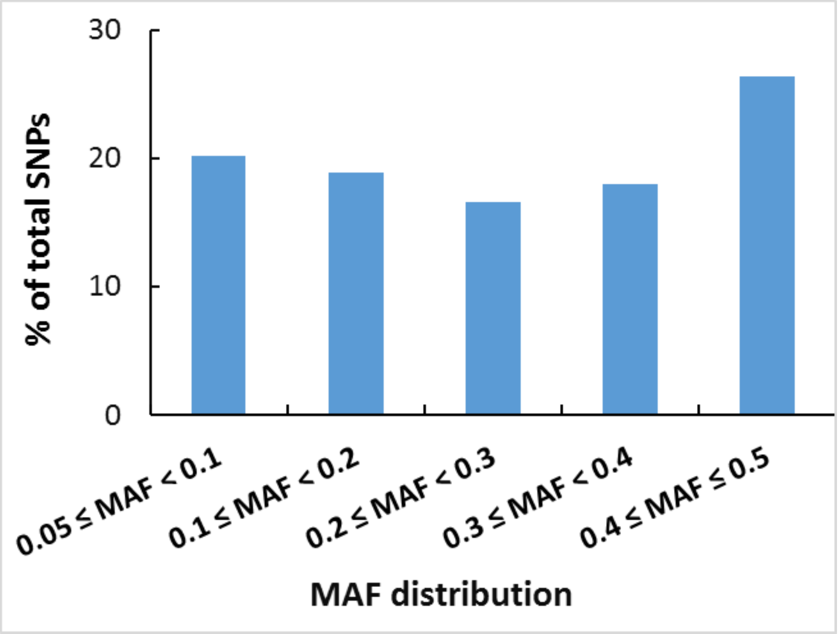

Figure S1: Distribution of minor allele frequency (MAF) of SNP loci detected in limber pine resistant gene analogs (RGAs).

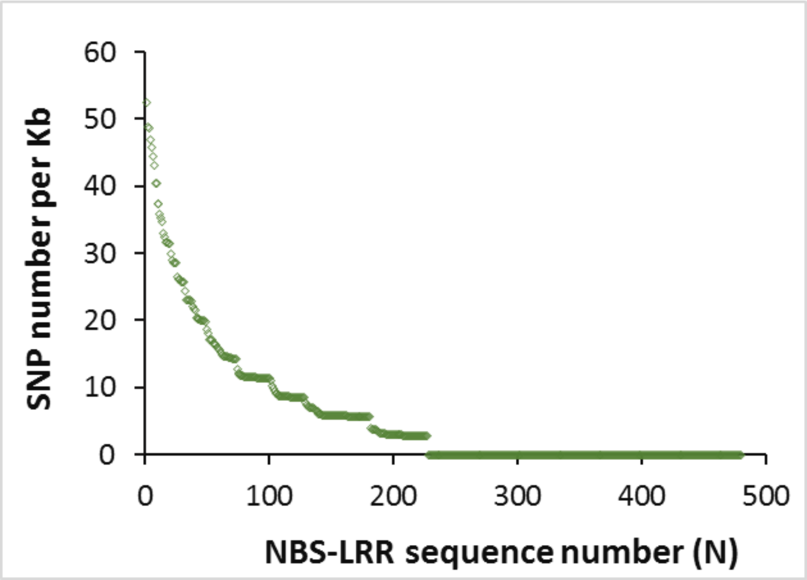

Figure S2: SNP frequencies of limber pine resistance gene analogs (RGAs).

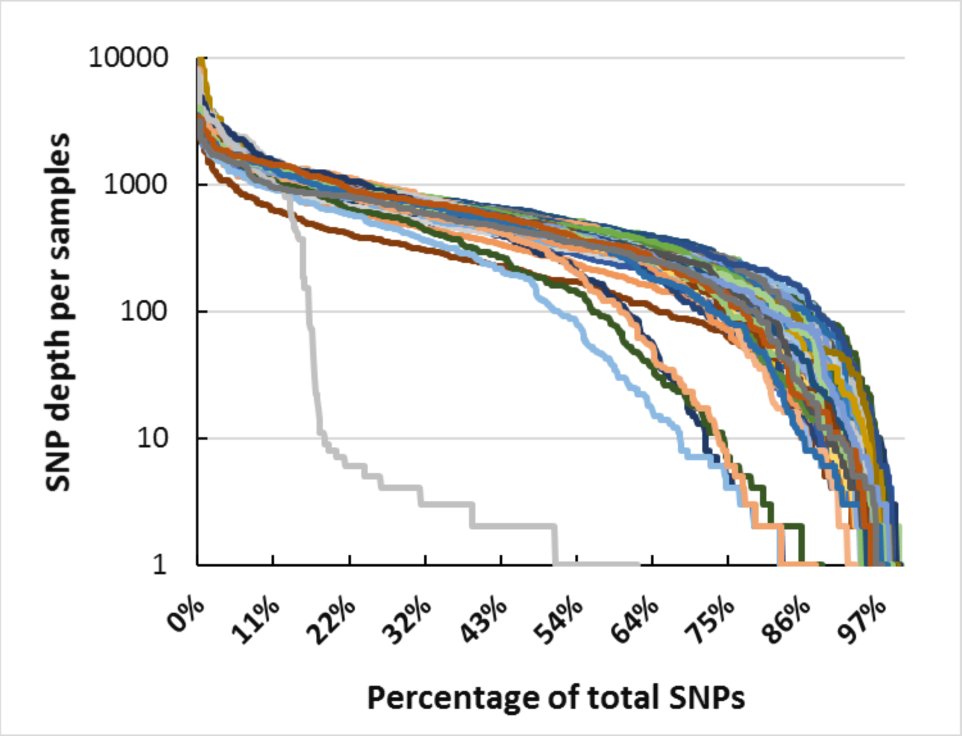

Figure S3: Sequencing depth of SNP loci in individual samples. The percentages of total SNPs (x-axis) have a coverage of a certain depth (y-axis). SNP depth was assessed in each individual of seed family LJ-112. The plot displays data for SNPs (967) with MAF ≥ 0.3 and about 70% of them have a minimum depth at 10 x in all samples except one.

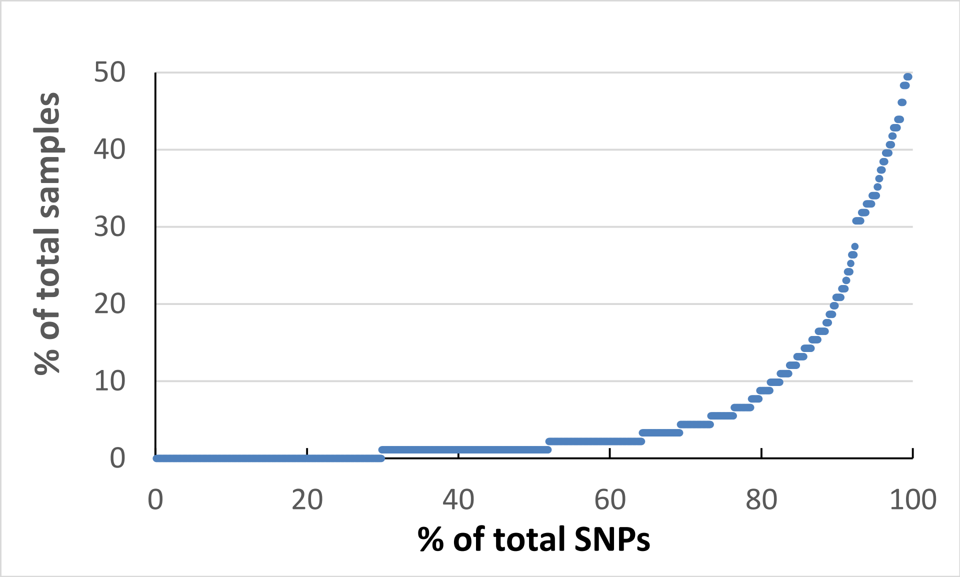

Figure S4: The extent of missing data for 967 SNP loci in 96 individual samples of seed family LJ-112. Individual samples with missing data were calculated as a percentage of the total (y-axis) and plotted across the cumulative total SNPs (x-axis). Over 80% of total SNPs showed missing data in less than 10% of total individual samples.

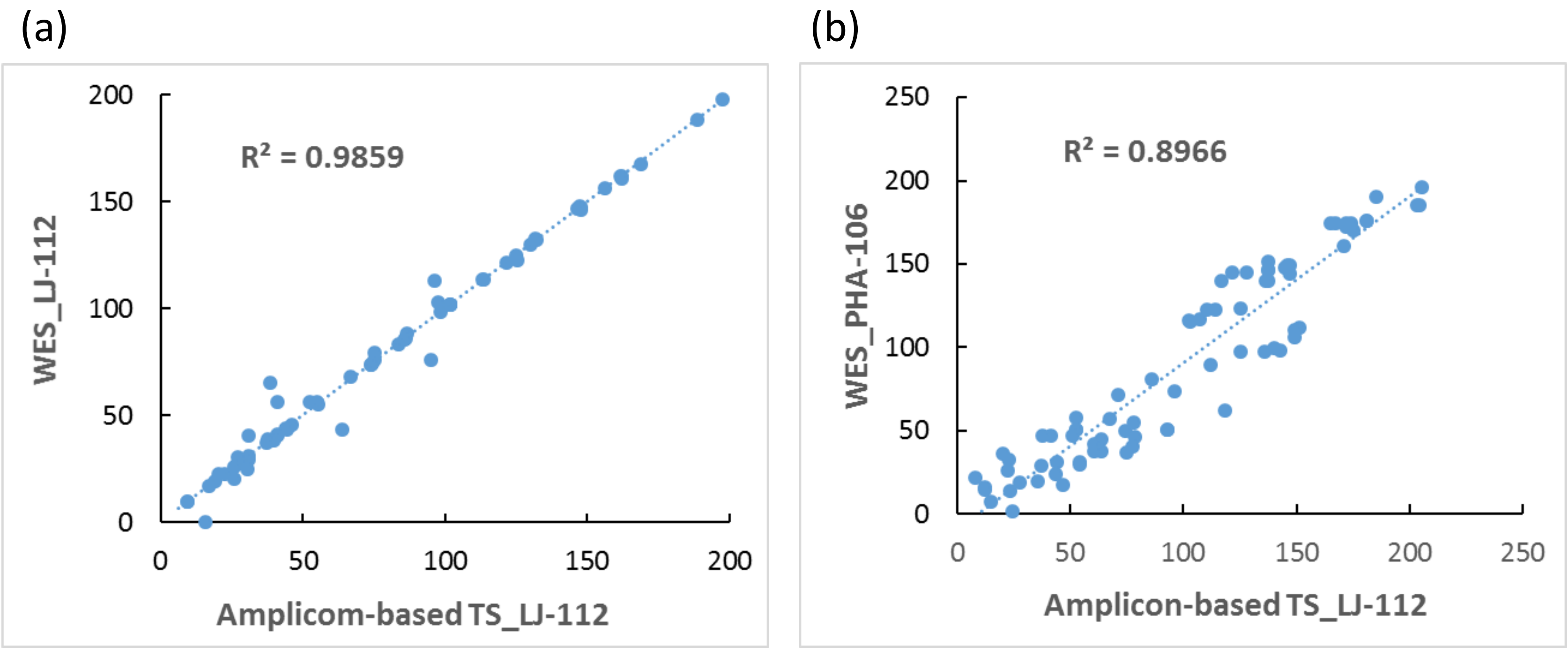

Figure S5: Correlation of SNP positions of the same NLR gene mapped on the same linkage groups (LGs) by both Fluidigm amplicon-bases targeted-seq (TS) and whole exome-seq (WES). (a) Comparison of TS and WES in seed family LJ-112; (b) comparison of TS and WES between seed families LJ-112 and PHA-106.

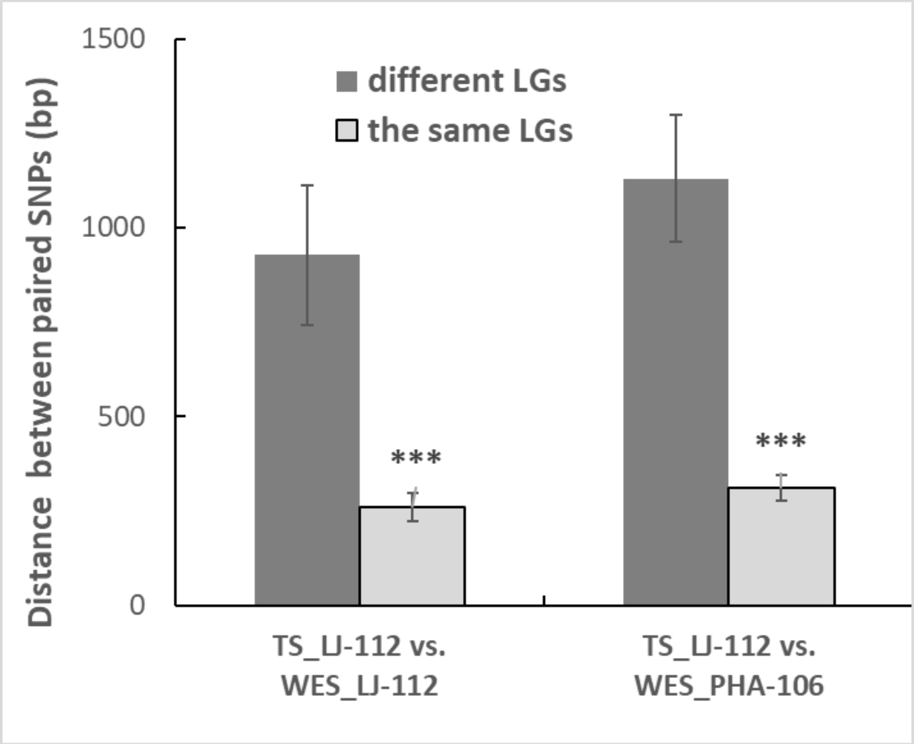

Figure S6: Physical distances (bp) of paired SNPs mapped by Fluidigm amplicon-bases targeted-seq (TS) and whole exome-seq (WES).

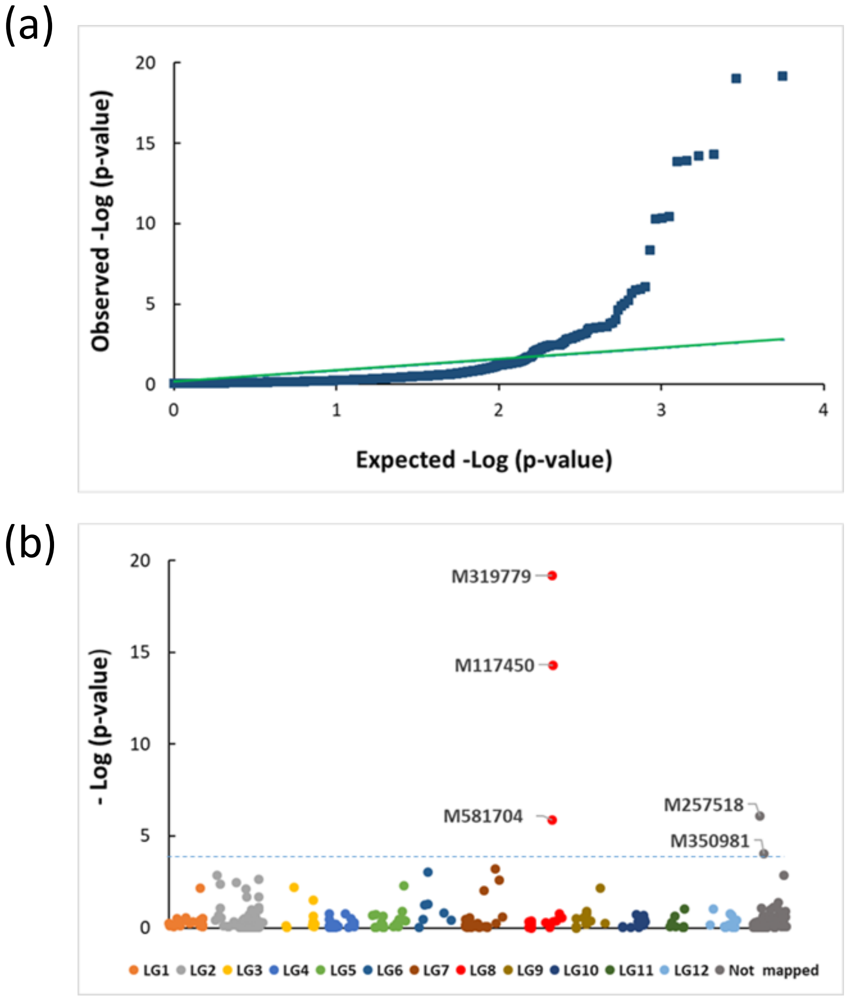

Figure S7:Identification of NLR alleles significantly associated with MGR-conferred phenotypes using extreme-phenotype genome-wide association study (XP-GWAS). (a) Quantile–quantile plot of the test statistic: 5,608 SNPs detected in 354 RGAs with coverage > 50 were subjected to association analysis. (b) Manhattan plot: top SNPs were selected for each genes and plotted against genetic maps of 12 linkage groups, and those genes not mapped so far were included as a separate group. Significant threshold value ($p = 1.69 \times 10^{-4}$) is presented by a horizontal dash line.
